# Sampling strategies for frequency spectrum-based population genomic inference

**DOI:** 10.1186/s12862-014-0254-4

**Published:** 2014-12-04

**Authors:** John D Robinson, Alec J Coffman, Michael J Hickerson, Ryan N Gutenkunst

**Affiliations:** Department of Biology, City College of New York, New York, NY 10031 USA; Current Address: South Carolina Department of Natural Resources, Marine Resources Research Institute, Charleston, SC 29412 USA; Department of Molecular and Cellular Biology, University of Arizona, Tucson, AZ 85721 USA; Subprogram in Ecology, Evolution and Behavior, the Graduate Center of the City University of New York, New York, NY 10016 USA; Division of Invertebrate Zoology, American Museum of Natural History, New York, NY 10024 USA

**Keywords:** Allele frequency spectrum, Demographic history, SNP, Model selection, Parameter uncertainty

## Abstract

**Background:**

The allele frequency spectrum (AFS) consists of counts of the number of single nucleotide polymorphism (SNP) loci with derived variants present at each given frequency in a sample. Multiple approaches have recently been developed for parameter estimation and calculation of model likelihoods based on the joint AFS from two or more populations. We conducted a simulation study of one of these approaches, implemented in the Python module δaδi, to compare parameter estimation and model selection accuracy given different sample sizes under one- and two-population models.

**Results:**

Our simulations included a variety of demographic models and two parameterizations that differed in the timing of events (divergence or size change). Using a number of SNPs reasonably obtained through next-generation sequencing approaches (10,000 - 50,000), accurate parameter estimates and model selection were possible for models with more ancient demographic events, even given relatively small numbers of sampled individuals. However, for recent events, larger numbers of individuals were required to achieve accuracy and precision in parameter estimates similar to that seen for models with older divergence or population size changes. We quantify i) the uncertainty in model selection, using tools from information theory, and ii) the accuracy and precision of parameter estimates, using the root mean squared error, as a function of the timing of demographic events, sample sizes used in the analysis, and complexity of the simulated models.

**Conclusions:**

Here, we illustrate the utility of the genome-wide AFS for estimating demographic history and provide recommendations to guide sampling in population genomics studies that seek to draw inference from the AFS. Our results indicate that larger samples of individuals (and thus larger AFS) provide greater power for model selection and parameter estimation for more recent demographic events.

**Electronic supplementary material:**

The online version of this article (doi:10.1186/s12862-014-0254-4) contains supplementary material, which is available to authorized users.

## Background

Population genetic data can be useful for comparing alternative representations of demographic history and for estimating parameter values under potentially complex models. The declining costs associated with next-generation sequencing, along with recent developments allowing multiple individual genomes to be simultaneously sequenced [1,2], have led to increases in the number of researchers generating genomic-scale datasets that include population-level samples of individuals. These datasets have the potential to provide unprecedented insight into the demographic history of populations and the evolutionary history of divergence among species [3]. Analyses based on the allele frequency spectrum (AFS) have become increasingly popular when considering population genomic datasets, in part due to the development of analytical software packages that consider the joint AFS between two or more populations [4-7].

The AFS is a *P*-dimensional array, where *P* is the number of populations considered, that gives the number of single nucleotide polymorphism (SNP) loci with derived alleles present at a given joint frequency in the sampled populations. Each dimension contains 2*n*_*i*_ + 1 elements, where *n*_*i*_ is the number of diploid individuals sampled from population *i*. These elements are ordered [0, 1, … , 2*n*_*i*_] along each dimension, and each value in the body of the array is the number of derived variants across the sample that are present at a given joint frequency. For instance, considering two populations, each SNP locus contributes one unit to the value in the AFS located at [*x*_*1*_, *x*_*2*_], where *x*_*i*_ is the number of derived allele copies (indexed on 0) in samples from population *i*. The joint AFS is based on these data, summed across the set of SNPs genotyped in two or more populations.

**Figure 1:**
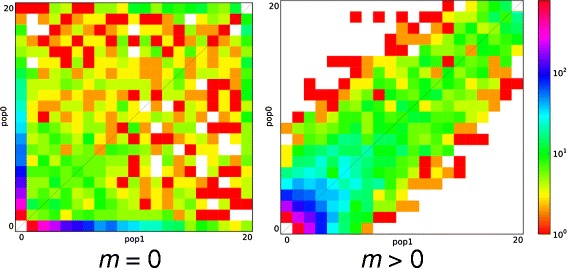
**Information in the allele frequency spectrum.** A comparison between two spectra of similar size (*n =* 10 diploid individuals sampled from each of two populations) that differ in the rate of migration between populations. Migration between populations increases the correlation in allele frequencies, thus increasing the density of SNPs falling along the diagonal of the AFS.

For datasets composed of biallelic, unlinked SNPs, the AFS is a complete summary of the data [4], and many commonly used statistics, such as the number of segregating sites, *F*_*ST*_, and Tajima’s *D* [8], can be calculated directly from the frequency spectrum. Additionally, patterns in the AFS can be indicative of demographic and/or selective events in the evolutionary history of the population or populations under consideration. For instance, gene flow between populations increases the correlation in allele frequencies, increasing the proportion of variable sites that fall along the diagonal of the AFS (Figure 1). The AFS is therefore well suited for the analysis of population genomic data, which are increasingly feasible to collect due to the rapid pace of development in sequencing technologies. Estimates of historical demography from the AFS can also be used to provide a baseline against which tests for the signatures of selection can be carried out [9-11]. However, the utility of parameter estimates obtained from analysis of the AFS will depend on their accuracy and precision, as well as the power of the analytical framework for model selection.

Several related computer programs have recently been introduced to analyze joint frequency spectra from two or more populations [4-7]. These programs differ in the specifics of their approach to modeling the AFS, using either diffusion approximation [4,6] or coalescent simulations [5,7] to model the density of SNPs in cells of the AFS. For comparisons between models and observed data, all of these methods employ composite likelihoods, which estimate the overall likelihood using combinations of likelihoods calculated from independent subsets of the data. For instance, in the context of the AFS, the composite likelihood is the product of the likelihoods calculated for individual cells of the spectrum. The similarities between software packages have resulted in similar performance of the different analytical methods in cases where they are directly compared [5-7], although some minor differences have also been noted [6]. Of these alternatives, δaδi [4] has been most widely applied, with applications to genomic data collected from humans [12-14], cattle [15], rice [16], and bees [17], among others.

Here, we use a simulation study to investigate the influences of sample size on the power for model selection and the accuracy of parameter estimates obtained from δaδi [4]. Because we employ an information-theoretic model selection approach, our use of the term power does not follow the standard statistical definition (the probability of rejecting the null hypothesis). Instead we define power as the probability of selecting the true (simulated) model from a set of competing candidates. Given the similarities between the AFS-based software packages discussed above, we expect that the results from our simulations will apply broadly to AFS-based methods for demographic inference. Several previous studies have investigated the accuracy of AFS-based parameter estimates and the power to detect historical demographic events using simulated data [4-7,18,19]. However, our analysis is differentiated from these assessments in that previous studies typically have not specifically explored the influences of the number of individuals sampled on the accuracy of estimates [4-7], or have simulated substantially smaller datasets [19]. Our analysis considers a broad range of one- and two-population models, and we investigate the influences of sample size on both the power for model selection and the accuracy of parameter estimates obtained from analysis of the AFS. We focus on the importance of the number of sampled individuals rather than the number of SNPs, as the former can be directly controlled during experimental design, while the latter is the result of a combination of the underlying history of the population under consideration, the size of the genome, and the sequencing effort expended. In the following, we therefore use the term sample size to refer to the number of individuals genotyped, rather than the size of the genomic region surveyed. Our results show that i) model selection and parameter estimation improve with larger sample sizes and ii) consistent with previous work [19,20], recent demographic events are more challenging and require substantially larger sample sizes for accurate inference.

## Results and discussion

**Figure 2:**
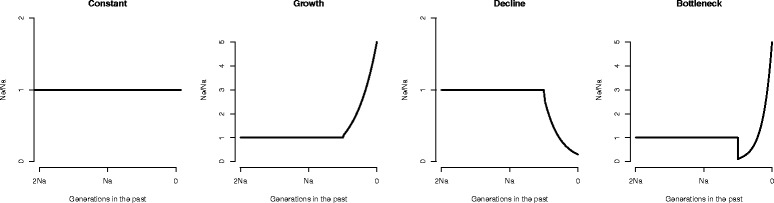
**Single-population demographic models.** Simulated models included a constant size model (SNM), a model of population growth (POSG), a model of population decline (NEGG), and a model involving a bottleneck followed by exponential growth (BG). Time moves from left (past) to right (present).

**Figure 3:**
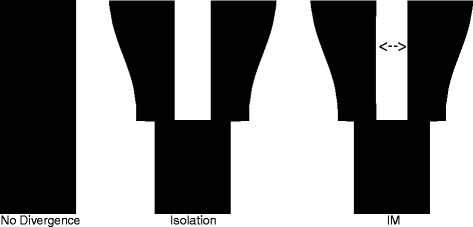
**Two-population demographic models.** Simulated models included a model of no divergence (SNM), a model of divergence in isolation (ISO), and a model of divergence with gene flow (IM). The width of the bar is proportional to population size through time, with time moving from the bottom (past) to the top (present) of the plot.

Our simulation study was designed to assess the influence of sample size (and thus the overall size of the AFS) on the power for model selection and the accuracy of parameter estimates obtained using diffusion approximations of the AFS for one- (Figure 2) and two-population models (Figure 3). We were also interested in the power of approaches based on the AFS to detect particular events or ongoing demographic processes. Thus, the candidate models included evolutionary processes or events that might be of interest in empirical studies [constant size (SNM) vs. population growth (POSG) vs. population decline (negative growth; NEGG) vs. a bottleneck followed by growth (BG); single population (SNM) vs. divergence in isolation (ISO) vs. divergence with gene flow (IM)]. We devised two parameterizations for each configuration, one where we expected the AFS to contain adequate information for likelihood evaluations (A; more ancient demographic events with strong patterns of population growth/decline) and another that was designed to be more difficult for parameter estimation and model comparison (B; more recent events with moderate growth/decline). We simulated 100 replicate datasets per sample size under each model considered, for each of these two parameterizations (Tables 1 and 2). The Akaike Information Criterion (AIC) [21], which includes a penalty for more highly parameterized models, and Akaike weights [22] were used to compare the relative fit of all candidate models to the simulated data (four models for single-population datasets, three models for two-population datasets). To assess the influences of sample size on the accuracy and precision of parameter estimates, we calculated the root mean squared error (RMSE) for each parameter in each model and examined estimates of uncertainty in individual parameter estimates obtained from the Hessian matrix (the matrix of second order partial derivatives of a function with respect to its parameters). We discuss our results for one- and two-population settings separately below, and conclude with sampling recommendations for future empirical studies and a consideration of the limitations of our simulation-based assessment of statistical power for demographic inference from the AFS.

**Table 1:** **Parameter values used in single-population model simulations**

**Parameter**	**Constant**	**Bottleneck**	**Growth**	**Decline**
*θ* ^a^	10,000	10,000	6000	29,000
*η*_*D*_	--	0.1/0.25	--	0.1/0.25
*η*_*G*_	--	5/2.5	5/2.5	--
*T*	--	0.25/0.025	0.25/0.025	0.25/0.025

Values are given for both A (ancient) and B (recent) parameterizations. Parameters are ancestral *θ*, the magnitude of population decline (*η*_*D*_) and growth (*η*_*G*_), and the time of the demographic event (*T*, in units of 2*N*_*A*_ generations, where *N*_*A*_ is the ancestral population size).

^a^:set to produce roughly equal numbers of SNPs across models for a given sample size under the A parameterization (~50,000 SNPs for samples of 100 chromosomes).

**Table 2:** **Parameter values used in two-population model simulations**

**Parameter**	**No Divergence**	**Isolation**	**IM**
*θ* ^a^	10,000	7250	7250
*s*	--	0.5	0.5
*η*	--	1	1
*T*	--	0.25/0.025	0.25/0.025
*m*_*12*_	--	--	1/10
*m*_*21*_	--	--	1

Values are given for both A (ancient) and B (recent) parameterizations. Parameters are ancestral *θ*, the proportion of the ancestral population founding population 1 (*s*), the ratio of current to ancestral size in each population (*η*), the time of the population split (*T*), and migration rates into population 1 from population 2 and vice versa (*m*_*12*_ *and m*_*21*_).

^a^:set to produce roughly equal numbers of SNPs across models for a given sample size (A ~ 50,000 SNPs for samples of 100 chromosomes).

### One-population models

#### Model selection

**Figure 4:**
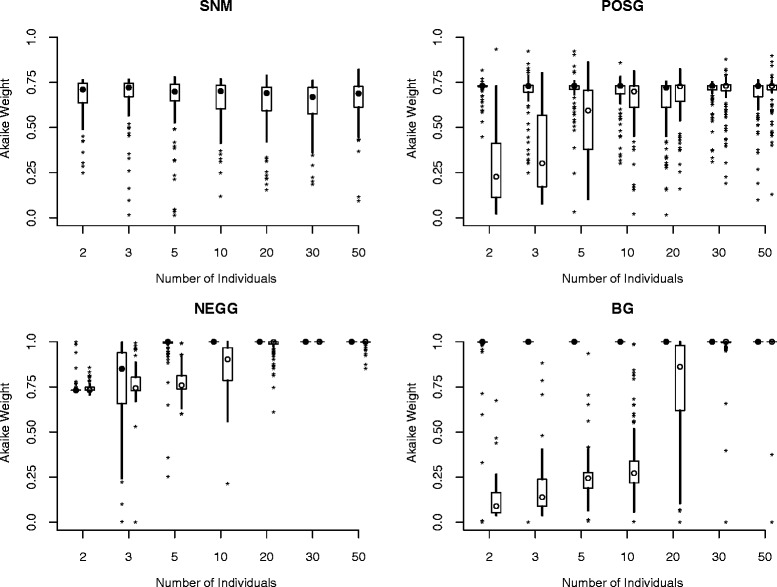
**Confidence in model selection for single-population models.** The distributions of Akaike weights in favor of the true model are shown as boxplots versus sample size. Both ancient (A, filled circles to the left) and recent (B, open circles to the right) parameterizations are shown. The circle represents the median Akaike weight, the box extends from the 25th percentile of the distribution to 75th percentile, lines include the range of values observed (minus outliers), and outliers (defined as values more than 1.5 times the interquartile range from the box) are plotted as asterisks.

For simulations of a single population, Akaike weights, which give the proportional support for each of the candidate models (summing to one), favoring the true (simulated) model increased with increasing sample size across most models and parameterizations considered, with some exceptions (Figure 4). These exceptions included the constant size model (SNM), the positive growth model (POSG) with ancient growth, and the bottleneck model (BG) with ancient growth. In these cases, median Akaike weights in favor of the simulated model were consistently high regardless of sample size (Figure 4). Interestingly, for the bottleneck (BG) model with recent growth (B parameterization), the true model typically was not strongly supported with sample sizes smaller than 20 diploid individuals. At these smaller sample sizes, the SNM model (*n* = 2 and 3 individuals) or the NEGG model (*n* = 3, 5, and 10 individuals) often received more support. However, there was very little separation in the log-likelihoods of the candidate models. In fact, the mean log-likelihood value of the BG model was the highest (by a narrow margin) of the candidate set across all simulated sample sizes. Thus, much of the support for the simpler models (SNM and NEGG) at smaller sample sizes resulted from the penalty imposed by AIC on the more complex BG model.

The improvement associated with increases in sample size was realized at smaller numbers of individuals for models with ancient demographic events. For instance, data simulated under the NEGG model were easily identified across sample sizes and parameterizations (Figure 4). Both recent and ancient declines were confidently identified (100% of Akaike weights *w*_*NEGG*_ > 0.85 in favor of the true model) in the largest sample sizes considered (*n* = 50). However, no replicate datasets with 10 or more individuals sampled had *w*_*NEGG*_ < 0.9 in the ancient population decline model, compared to 59 replicates (out of 400 datasets) with a more recent population decline. This illustrates the challenge posed for accurate model selection when considering recent demographic events.

Our results were encouraging for model selection based on the AFS, even for small samples in terms of the number of individuals genotyped. However, if demographic changes are very recent, the power for model selection is reduced, and large samples, in terms of both the number of individuals and the number of SNPs, may be required to confidently choose among competing demographic models [19,20]. Additionally, with extremely small sample sizes, the log-likelihood values calculated from the AFS are relatively small in magnitude (with the product of likelihoods across the AFS calculated from fewer cells). Our results raise caution for application of penalized AIC in these cases, as the penalty imposed on more complex models may be sufficient to obscure substantial support from spectra with few total cells.

#### Parameter estimation

Median parameter estimates from δaδi [4] converged to their true values (across all models and both parameterizations) as sample size increased, indicating that the parameter estimates were unbiased (Additional file 1: Table S1). Across simulated models and sample sizes, the parameter estimates for most models, when fit to their simulated datasets, converged by our criterion (i.e., three model fits within 5 log-likelihood units of the best fit) in fewer than 10 iterations. However, the more parameter-rich BG model with recent population growth and large samples (*n* = 50) required an average of more than 26 fits to converge. Interestingly, fits of the “wrong” models (e.g. fitting a model of population growth to data simulated under a decline) also required additional iterations for convergence.

**Figure 5:**
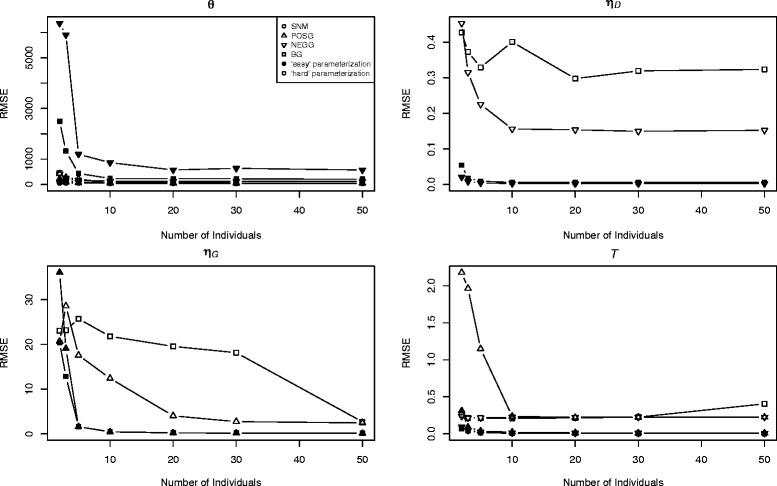
**Accuracy of parameter estimates for single-population models.** Plots show RMSE versus sample size for each of the four single-population models. Both ancient (A, filled circles to the left) and recent (B, open circles to the right) parameterizations are shown.

We used the root mean squared error (RMSE) to assess the accuracy and precision of parameter estimates for the simulated datasets. Parameter estimates for single-population models generally improved (RMSE declined) with larger samples across the models and sample sizes simulated (Figure 5). As expected, RMSE for parameters in simulations with recent demographic events were often larger than for data simulated under the same model with more ancient demographic events (Figure 5). Overall, the gain in precision and accuracy associated with sampling more individuals was subject to diminishing returns with the RMSE tending to level off above *n* = 5 to 10 for ancient events and above *n =* 10 to 20 for more recent demographic events (Figure 5). Nonetheless, some improvement in parameter estimates (e.g., *η*_*G*_) could be seen as sample size increased to 50 diploid individuals under the more complex models (e.g., the BG model; Figure 5). In most cases, our results suggest that samples of 10 to 20 diploid individuals may be sufficient for AFS-based demographic analysis of single-population genomic datasets. However, substantially larger sample sizes may lead to additional improvements in the accuracy and precision of parameter estimates from the AFS (see Extreme Cases below).

As a second way to quantify parameter estimation performance, we used the Hessian matrix, which is the matrix of second-order partial derivatives of a function with respect to its parameters, to construct 95% confidence intervals (CI) around the maximum-likelihood point estimates for the parameters of our models. In agreement with trends in the RMSE, analyses of the Hessian matrix demonstrated that uncertainty declined (producing narrower confidence intervals) for larger sample sizes, with the improvement in CI widths subject to diminishing returns as sample size increased (Additional file 1: Table S1). For ancient demographic events, much of the improvement in the width of 95% CI was seen in samples as small as *n* = 20. In contrast, Hessian uncertainties for parameters of models with recent events declined up to the largest population sizes simulated (Additional file 1: Table S1), suggesting that further increases in sample size might lead to additional improvements in the widths of 95% CI.

We further quantified the accuracy of inferences drawn from the AFS by examining the coverage property of CI calculated from our parameter estimates and their associated uncertainties. Coverage often increased with sample size, with three of the four models showing adequate coverage for all parameters at the largest sample sizes simulated (Additional file 1: Table S1). However, there were some cases of poor performance of confidence intervals. For instance, coverage of 95% CI for *θ* in the BG model with an ancient bottleneck was lowest at the largest sample size simulated (67% at *n* = 50). With more ancient events and excluding the BG model, 95% CI performed well, containing the true parameter value for all model parameters in more than 90% of replicates for datasets composed of 10 or more diploid individuals (Additional file 1: Table S1). For the BG model, both recent and ancient bottlenecks led to poor performance, and 95% CI contained the simulated value in far fewer replicates than expected. The poor performance of the CI for the BG model parameters may be the result of a combination of estimated CI that were too narrow and imprecision in parameter estimates that was exacerbated with more recent demographic events. All statistics associated with parameter estimates obtained for single-population models (median parameter estimates, median uncertainties, RMSE, and proportionate coverage of 95% CI) are given in Additional file 1: Table S1.

### Two-population models

#### Model selection

**Figure 6:**
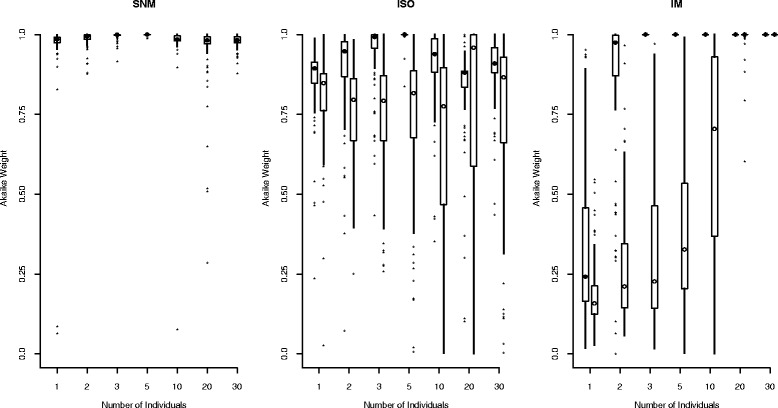
**Confidence in model selection for two-population models.** The distributions of Akaike weights in favor of the true model are shown as boxplots versus sample size. Both ancient (A, filled circles to the left) and recent (B, open circles to the right) parameterizations are shown. Boxplots are constructed as in Figure 4.

Similar to the pattern observed for simulations of single-population models, Akaike weights in favor of the true model generally increased with increasing sample sizes across the simulated two-population models (Figure 6). An exception to this trend was seen for the ISO model with recent divergence, which had median Akaike weights > 0.75 across all sample sizes considered. For both the ISO and IM models, Akaike weights in favor of the true model at any given sample size were typically higher for models with ancient population divergence than for those with recent divergence. For example, under the IM model, the median Akaike weight reached 1.0 (100% support for the true model) at a sample size of *n* = 3 per population for models with more ancient divergence. Similar performance was not realized for recent divergence until sample sizes of *n* = 20 per population (Figure 6). Furthermore, the distribution of Akaike weights under the more recent models of divergence (with or without migration) indicated that there was substantial support in favor of alternative models, even at relatively large sample sizes (Figure 6). For example, the interquartile range (25th – 75th percentile) of Akaike weights for the ISO model with recent divergence included values less than 0.5 with *n* = 10 per population (Figure 6). The very recent nature of divergence in the B parameterizations was likely responsible for this trend, as models with moderate gene flow and strict isolation may not be identifiable at recent divergence. More generally, the ability to differentiate between divergence models with and without gene flow will decrease as the divergence time (*T*) and/or the migration rate (*m*) go to zero. Due to problems achieving convergence of ML estimates for the IM and ISO models fit to some simulated datasets under our criterion (i.e., three optimizations within five log-likelihood units of one another), our assessment of model selection performance was limited to a slightly smaller number of total replicate datasets to which all models were successfully fit (see Methods for additional details).

#### Parameter estimation

**Figure 7:**
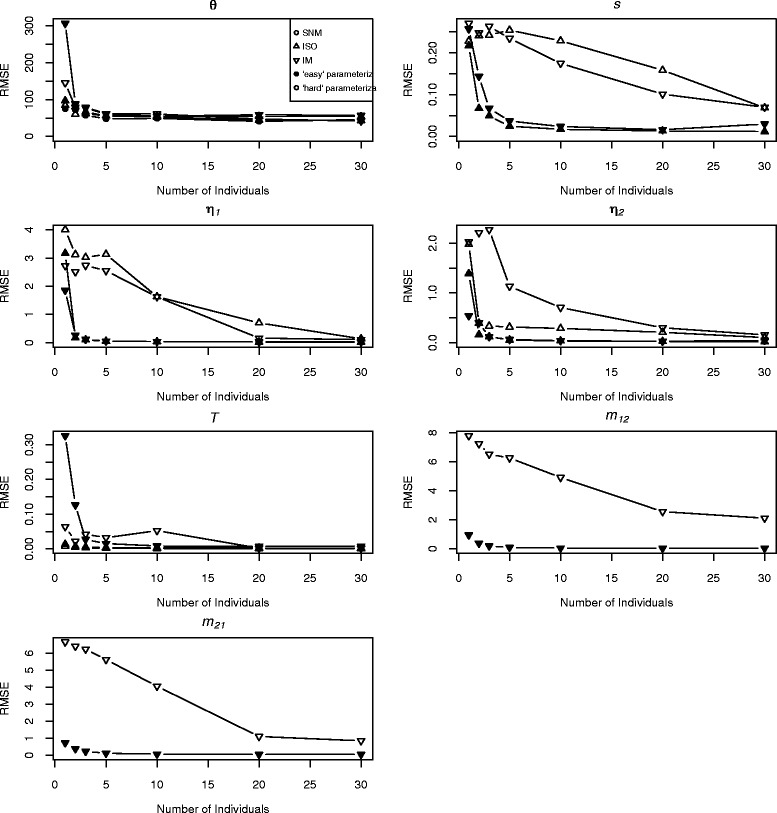
**Accuracy of parameter estimates for two-population models.** Plots show RMSE versus sample size for each of the three two-population models. Both ancient (A, filled circles to the left) and recent (B, open circles to the right) parameterizations are shown.

As in the single-population simulations, the accuracy and precision of parameter estimates improved with larger sample sizes, for both recent and ancient population divergence models (Figure 7). Two-population models also converged more slowly to their maximum likelihood estimates when fit to data simulated under alternative models. For instance, fitting the IM model to data simulated under the ISO model with ancient divergence required, on average, more than 14 iterations for samples of 10 diploid individuals per population. Parameter estimates were largely unbiased for the parameters and models considered and, in most cases, they converged to their simulated values in the larger sample sizes (Additional file 1: Table S2).

One exception was seen in the estimates of parameters associated with population size changes during divergence (*s, η*_*1*_, and *η*_*2*_) in datasets simulated under the ISO model with recent divergence. In these cases, *s* was subject to a downward bias for all but the largest sample sizes, while *η*_*1*_ and *η*_*2*_ were biased high and low, respectively (Additional file 1: Table S2). These biases are likely related to the starting points used for optimization, the perturbation settings for the *s* parameter, and a flat likelihood surface. More specifically, the combination of the starting point for *s* (0.25) and the perturbation setting (within 1-fold of starting point) caused the optimizer to only be initialized with *s* between 0.125 and 0.5. Moreover, the optimizer was not driven to explore larger values of *s* because the likelihood surface is very flat with respect to *s* for data simulated under very recent divergence. We refit these data using a starting point of *s* = 0.5 and allowing perturbation within 2-fold around this point. These revised settings eliminated the downward bias originally seen in *s* estimates for these datasets (Additional file 1: Figure S1). Median estimates of *s* were between 0.46 and 0.53 for all sample sizes. In practice, we suggest that perturbation settings be chosen to ensure that the starting points for optimization of model parameters extend beyond the range of values that the data constrain the model to. For empirical datasets, if optimization end points result in very similar likelihood values, even with very different parameter estimates, the likelihood surface may be flat over the region explored during optimization. In these cases, a broader range of starting points may help to correct potential biases in optimized parameter estimates.

For datasets with small sample sizes and thus large parameter uncertainties, inferred values of *s, η*_*1*_, and *η*_*2*_ were correlated, suggesting that an acceptable “ridge” of parameter space exists, along which the parameter estimates associated with the proportion of the ancestral population that founded a derived population and the magnitude of expansion in that new population were correlated. For example, there were strong correlations in opposite directions between the estimated values of *s* and both *η*_*1*_ (Pearson’s r = −0.56; p << 0.001) and *η*_*2*_ (Pearson’s r = 0.57; p << 0.001) for datasets with 10 diploid individuals per population. This pattern is consistent with a range of possible divergence/growth scenarios that are each able to fit the data approximately equally well. This issue is not unique to δaδi [4], as previous authors have noted problems distinguishing alternative demographic histories with data from the AFS [23]. An additional (downward) bias was noted for estimates of *m*_*21*_ for the largest datasets simulated (*n* = 20 and 30 per population) under the IM model with recent population divergence (Additional file 1: Table S2). Aside from these instances, median estimated parameter values approached their true values for sample sizes as small as *n* = 3 per population (Additional file 1: Table S2).

RMSE for parameter estimates under models with recent divergence indicated that substantially larger sample sizes were needed for accurate parameter estimation in cases of recent isolation (Figure 7). For instance, for models that included ancient population divergence, much of the gain in accuracy and precision was achieved at relatively small sample sizes (*n* = 2 to 3 per population). However, with recent divergence RMSE values for some parameters declined steadily across the sample sizes simulated (e.g., *s* and *m* for the IM model; Figure 7). Thus, it seems likely that parameter estimation for recent divergence models would continue to improve beyond the largest sample sizes simulated in our study.

In the IM model with recent divergence (B parameterization), we included asymmetric migration between populations, which is known to result in bias when employing approaches that assume symmetry in migration rates [24-26]. Interestingly, the direction of gene flow asymmetry (with point estimates of *m*_*12*_ > *m*_*21*_) was recovered in more than 80% of the replicates across simulated sample sizes. Given no useful information for estimation of these parameters, we would expect that approximately 50% of replicates would have estimated *m*_*12*_ larger than *m*_*21*_. Thus, it would seem that even when the parameter estimates and model likelihoods were largely unreliable (i.e., for very small sample sizes), useful inference could still be gained for the direction of gene flow asymmetry. Nonetheless, the CI for the two migration parameters overlapped in almost all instances for smaller sample sizes, indicating that the power of the method to confidently infer asymmetric migration was limited, particularly with recent divergence and small samples. By contrast, with *n* = 20 or 30 per population, confidence intervals for these two parameters often did not overlap, and the direction of asymmetry could be confidently inferred (in 88% and 96% of simulated datasets, respectively).

Hessian estimates of uncertainty declined precipitously with increasing sample size across models for two-population simulations with ancient divergence (Additional file 1: Table S2). As was the case for RMSE, much of the improvement in the uncertainty occurred as sample size reached three to five individuals per population. The more parameter-rich IM model tended to have larger uncertainty estimates for a given parameter at each sample size than did the ISO model (Additional file 1: Table S2), as might be expected given the difference in the number of parameters estimated from the data for these two models (seven for IM, five for ISO). As seen for the single-population models, coverage rates of 95% CI, based on Hessian estimates of uncertainty, were often lower for parameterizations involving recent population divergence than for those with ancient divergence (Additional file 1: Table S2). For models involving older divergence, 95% CI performed relatively well, with true parameter values covered by CI at least 84% of the time for sample sizes of two diploid individuals or more per population. Similar performance was noted for data simulated under recent divergence models, for at least some parameters (*θ*, *T*), while 95% CI for others (*s, η, m*) displayed markedly lower coverage rates, resulting from a combination of parameter estimation bias (as mentioned above) and underestimation of the width of CI. All statistics associated with parameter estimates obtained for two-population models (median parameter estimates, median uncertainties, RMSE, and percent coverage of 95% CI) are given in Additional file 1: Table S2.

### Extreme cases

In order to more fully explore the influence of sample size and the timing of demographic events on inferences drawn from the AFS, we conduced three sets of additional simulations with parameter values and sample sizes more extreme than those considered in our full simulation study. For the single-population models explored, we included simulations under the A model parameterization but with substantially older demographic events (*T* = 1.5; events occurring 3*N*_*A*_ generations ago, where *N*_*A*_ is the ancestral effective population size). We expected that these datasets would be more difficult for parameter estimation and model selection, due to the substantially more ancient timing of the simulated events. We also further explored the influence of sample size in single-population models by simulating samples of 1351 diploid individuals, as in the joint AFS analyzed by [27]. Finally, we tested the performance of AFS-based inference for two-population models with extremely recent divergence (*T* = 0.005). Parameters for these additional simulations closely followed the A parameterization (Table 1), but *θ* was altered to produce similar numbers of SNPs (Additional file 1: Tables S3-S5).

To verify that the AFS from the diffusion approximation implemented in δaδi matched expectations, we compared the AFS from δaδi with those obtained using the coalescent simulator ms [28] for the extreme cases we considered. For the single-population models, the AFS simulated in δaδi matched that from ms [28] quite closely across the range of frequency categories for both ancient growth with moderate sample size (T = 1.5; *n* = 30; Additional file 1: Figure S2) and more recent growth with extremely large sample size (T = 0.25; *n* = 1351; Additional file 1: Figure S3). Furthermore, Anscombe Poisson residuals indicated no systematic bias across entries in the AFS for 30 individuals (Additional file 1: Figure S2). For the simulations involving large sample size, δaδi overestimates the number of SNPs in low frequency categories (Additional file 1: Figure S3). As a *post hoc* assessment of the influence of this overestimation on parameter estimates from δaδi, we generated 100 replicate frequency spectra in ms [28] under the same model (population growth), parameter values, and sample size (*n* = 1351). Each replicate dataset simulated in ms was generated by combining 2300 simulated loci (without recombination within a locus) with *θ* = 1 (to match *θ* in Additional file 1: Table S4). These datasets were then analyzed in δaδi to obtain maximum likelihood estimates for the parameters of the growth model. Similar to the overall pattern observed for single-population spectra, simulated frequency spectra for a model of very recent population divergence (*T* = 0.005) with a moderate sample size (*n* = 30 per population) agreed closely between ms and δaδi (Additional file 1: Figure S4), but with slight overestimation of the number of rare variants falling along the diagonal of the joint AFS and the opposite pattern for rare alleles with more divergent frequencies.

Our simulations of older demographic events resulted in strong support in favor of the simulated model for three of the four models compared (Additional file 1: Figure S5). Median Akaike weights from these datasets agree with previous results in that the bottleneck and growth models show substantial overlap in Akaike weights. In fact, data simulated under the bottleneck model often showed more support for the exponential growth model (Additional file 1: Figure S5). Parameter estimates for the POSG model in these cases suggest that a much older and larger expansion could produce similar patterns in the AFS. Median parameter estimates for the POSG model fit to these datasets were *T* = 4.3 and *η*_*G*_ = 19, much larger than the simulated values of 1.5 and 5, respectively. Parameter estimates obtained for fits of the correct model show substantially larger RMSE for some parameters than similarly sized datasets with more recent divergence (e.g., *T,* η; Additional file 1: Table S3). Thus, as expected, the quality of inferences obtained from the AFS is reduced as demographic events become much older.

Datasets simulated with larger sample sizes showed that, while our original simulations exhibited a pattern of diminishing returns with increasing sample sizes, there is still substantial room for improvement for all models if very large samples are collected. For the larger sample sizes (*n* = 1351), median Akaike weights in favor of the true (simulated) model were higher in each case than for the competing candidate models (Additional file 1: Figure S6). Some overlap was evident in weights for the POSG and BG models, when fit to data simulated under a model of exponential growth (POSG). In these cases, parameter estimates for the BG model closely matched those for the POSG model, with *η*_*D*_ estimated near 1.0 (median estimate = 0.85). Notably, the POSG model is a special case of the BG model where *η*_*D*_ = 1, thus the good fit of the BG model in this case is not unexpected. RMSE values for parameter estimates, given these large datasets, show improvement over those from the second largest datasets simulated in all but two cases (*θ*_*SNM*_ and *η*_*NEGG*_), and in no cases were RMSE values substantially larger than in previous simulations (Additional file 1: Table S4).

The overestimation of SNP densities in rare frequency categories noted in the comparisons of simulated spectra does not appear to bias results from δaδi. Median parameter estimates for ms-generated datasets were very close to the simulated values in all cases (median estimates: *θ* = 2295, *η*_*F*_ = 5.015, *T* = 0.246) and parameter RMSE was only slightly elevated above that for datasets simulated in δaδi (RMSE: *θ* = 36.3, *η*_*F*_ = 0.085, *T* = 0.015; compare with values in Additional file 1: Table S4). Thus, the minor discrepancies between ms and δaδi did not seriously impact the estimation of parameters for ms-generated datasets, suggesting that parameter estimates obtained for empirical datasets should be unbiased.

Finally, we also simulated datasets with much more recent divergence between two populations (*T* = 0.005) under the largest sample sizes considered in our previous simulations (*n* = 30 per population). Given the reduced performance of our simulations as *T* declined from 0.25 to 0.025, we expected that these additional simulations would show more uncertainty in model selection and larger RMSE for parameter estimates. Model selection results showed that, even at such recent divergence, the SNM and ISO models could be confidently identified (Additional file 1: Figure S7). However, the data simulated under the IM model often gave more support to the ISO model, probably as a result of the penalty imposed by AIC on the IM model (as in the BG model with small sample sizes, discussed above). In 76 of the 100 simulated datasets, raw likelihood values were greater for the IM model, by an average of just over one log-likelihood unit. Parameter estimates were also more accurate and precise for the simpler models, with no evidence of severe biases for parameters of the SNM and ISO models. By contrast, in the IM model, median estimates of parameters associated with population expansion (s, *η*_*1*_, *η*_*2*_) and migration (*m*_*12*_ and *m*_*21*_) deviated substantially from their simulated values (Additional file 1: Table S5). Parameter RMSE calculated for the simpler models were generally comparable to those for recent (*T* = 0.025) divergence models in similarly sized datasets (Additional file 1: Table S2). The time of divergence between populations (*T* = 0.005) was accurately estimated for both the IM and ISO models; estimates for this parameter ranged from 0.0043 to 0.0057 across the 100 simulated datasets. Thus, while the recent nature of divergence led to inaccuracy in estimates of several parameters, the performance of δaδi was better than expected for these datasets. Given the results presented above for single-population datasets, it seems likely that larger sample sizes (beyond 30 diploid individuals per population) would also improve parameter estimates in cases of very recent divergence.

### Sampling recommendations

Our results show that optimal sampling strategies for AFS-based inference are very much dependent on whether the underlying, and inherently unknown, evolutionary history involves recent or ancient demographic events. For instance, for projects examining patterns of divergence between populations or species with a long history of separation relative to their expected effective population sizes (e.g., geminate species pairs separated by the Isthmus of Panama), large numbers of individuals may not be required. However, if interest lies primarily in estimating migration rates among populations recolonized since the last glacial maximum, substantially larger sample sizes may be necessary. Based on our results, for demographic events occurring on the order of 0.5*N*_*A*_ generations ago, samples of *n* = 5 for single-population datasets, and *n* = 3 per population for two-population datasets (as previously noted [29]), would appear to be sufficient for accurate model selection and parameter estimation. On the other hand, if the sampled population has been subject to very recent or very ancient demographic events, larger samples will be necessary for confidence in model selection and parameter estimation. The influence of timing seen here is in agreement with previous work [19,20]. For instance, thousands of individuals may be required to detect the history of recent, explosive growth in the global human population [20].

Despite the importance of event timing on the performance of AFS-based analyses, reasonable results could be obtained, even for the recent events simulated here, given moderate sample sizes. For single-population models with recent demographic events (*T* = 0.025), samples of 20 or more diploid individuals resulted in the best performance for parameter estimation and model selection (Additional file 1: Table S1). Similarly, parameters of the two-population ISO and IM models were most accurately estimated (with minimal RMSE and reasonable coverage of 95% CI) at the largest sample size simulated (*n* = 30 per population). The IM model did show lower coverage of confidence intervals, but RMSE for all shared parameters was comparable between the IM and ISO models with recent divergence at the largest sample sizes (Additional file 1: Table S2). Furthermore, simulated datasets with *T* = 0.005 produced remarkably accurate estimates of *T*, despite bias in estimates of migration and expansion parameters. Thus, our study shows that useful inferences can be gained through analysis of the AFS, even for very recent demographic events and moderate demographic changes, given population-level samples of individuals.

### Caveats

The results presented above may be somewhat conservative for the ability of the AFS to distinguish between alternative models. For instance, by setting the upper bound for optimization of the parameter *η*_*D*_ at 1.0 for the BG model, our analysis allows the BG model to fit data simulated under the POSG model (exponential growth without a population bottleneck). Similarly, ISO and IM models with very recent times of divergence should provide good fits to data simulated under the SNM model (i.e., without divergence). In other words, there are inherent model overlaps and identifiability dynamics that may allow researchers to obtain useful parameter estimates even if one selects the wrong model. Furthermore, it is likely that Akaike weights would improve if the bounds for parameter optimization were more tightly constrained for key parameters. Limiting the estimation of the bottleneck severity parameter (*η*_*D*_) in the BG model to smaller values would likely serve to increase the Akaike weight in favor of the POSG model, when comparisons are made in data simulated under the latter history. With this limitation, the BG model would be forced to include a substantial bottleneck, rather than allowing the population to maintain near constant size before the initiation of exponential growth.

The use of composite likelihoods in AFS-based inference assumes that SNP loci in the sample are independent and unlinked. Strictly speaking, this is an unrealistic assumption, as physical linkage between sites, particularly those located on the same genomic fragment (e.g., in short-read datasets produced by RADseq; [1]), is a certainty. However, composite likelihoods have been shown to be consistent estimators across a range of population models [30]. While the expectation of the AFS is accurately recovered using composite likelihoods, ignoring linkage among sites results in underestimating the variance of the AFS [4]. Therefore, parameter estimates should be unbiased, but associated confidence intervals are not reliably calibrated [3] and the support for the best model may be overestimated in model comparisons based on composite likelihoods [7]. This bias should not affect our results, as our simulated data meet the key assumption of SNP independence. For empirical datasets collected from natural populations, bootstrap replicates can be used to approximate confidence intervals around parameter estimates [4], and modifications to AIC can help correct for biases in model selection [31].

Our simulations ignored the influences of selection on model selection and parameter estimation from the AFS. In empirical datasets, the inclusion of SNP loci under selection could bias results in a variety of ways, depending on the nature of selection acting on the loci. For instance, selective sweeps would remove variation from the population and leave similar patterns to those expected after expansion from a bottleneck. In practice, the underlying demography can be estimated from a putatively neutral subset of the AFS (e.g., third codon positions or synonymous mutations), assuming that hitchhiking via linkage to selected regions has not affected SNP frequencies of the subset. Then, demographic parameter estimates from this analysis can be used to set critical values for selection scans to identify SNP loci that deviate from expectations [9-11,18]. However, if the primary goal of the analysis is to estimate population demography, ignoring the influences of selection could result in biased estimates for demographic parameters. This may be more of a concern with widespread purifying selection in the genome than for selective sweeps at a limited number of loci, as the latter would likely result in less overall bias.

Our recent parameterizations were designed to pose substantial difficulty to inference from the AFS. As such, the recent parameterizations for single-population models included more relaxed population size changes, but the same *θ* values used for models with more ancient demographic events. Thus, the influence of the timing of events on model selection and parameter estimation is confounded with the severity of the demographic changes modeled and the number of SNPs in the sample (Additional file 1: Table S6). It is likely that the power for model selection would increase given more severe bottlenecks or greater population growth, even in models with very recent demographic events.

It is important to note that the quality and reliability of demographic parameter estimates from the AFS are inherently linked to the quality of the genotype calls resulting from the sequencing technology employed. In particular, low-coverage genomic data may pose problems for inference from the AFS. In these datasets, sufficient coverage may not be available to confidently identify variable sites and exclude sequencing errors. If not properly accounted for, these errors can lead to inaccurate genotype calls and biased allele frequency estimates, reducing the reliability of demographic inferences drawn from these data [32]. For instance, simulated datasets (including sequencing error) showed a bias toward more recent population growth in the AFS from low-coverage (4x) genomic data [33]. Nonetheless, methods have recently been developed to directly infer the AFS, without the need for SNP or genotype calling, from next-generation sequence data [34]. Such methods may provide a more robust estimate of the AFS for low-coverage genomic sequencing datasets and eliminate potential biases in demographic inferences from these data.

In general, simulation studies are blunt instruments for the assessment of statistical methods [35]. Thus the applicability of the results from our simulation study is fundamentally limited by our range of demographic models and sample sizes simulated. Furthermore, our analysis is based on a limited number of replicates per model and parameter combination (100 replicate datasets), making our estimate of performance a Monte Carlo approximation given finite computational resources. Our requirement that three parameter optimizations produced model likelihoods within five log-likelihood units of the maximum likelihood (see Methods) greatly increased the computational burden associated with parameter optimization. When fitting the BG model to datasets simulated with a recent bottleneck and 50 individuals sampled, this requirement led to an average of more than 26 optimizations per dataset (up to a maximum of 86 separate optimization steps). The number of parameter optimizations necessary to achieve convergence under this criterion became even larger in other cases. For the BG model fit to data simulated under an ancient exponential population decline (NEGG model, A parameterization) with *n* = 50, convergence required an average of more than 323 optimizations per replicate (up to a maximum of 1011 optimizations). Generally, long convergence times may indicate a poor fit of the model to the data, as (with the exception of the BG model mentioned above) convergence for the simulated model was typically faster. Other approaches, which coarsen the AFS [5-7] by combining entries, may converge more reliably, but such coarsening may also reduce statistical power.

## Conclusions

Previous studies have found that large sample sizes are required for confidence in model selection and parameter estimation given recent population growth [19,20]. However, the influence of sample size was model-dependent. For instance, increasing sample size above 3 diploid individuals per population did not substantially affect demographic parameter inference in a model of population divergence in isolation [29]. The results from our simulation study agreed with previous work focusing on the impacts of sample size and/or the timing of demographic events on the accuracy of inferences drawn from analysis of the AFS [5,18,19], in that more ancient demographic events (A parameterizations) typically allowed for increased confidence in model selection and parameter estimation. However, very old demographic events (on the order of 3*N*_*A*_ generations ago) also posed difficulty for inference based on the AFS, as the signal of a past demographic event is lost as populations approach mutation-drift equilibrium. Generally, improvements in both model selection and parameter estimation analyses were subject to diminishing returns as sample size increased, but much larger sample sizes still led to more accurate parameter estimates and model selection. Thus, when large sample sizes are attainable, more complex models involving much more recent demographic events may still be successfully analyzed using the AFS. While our simulation study is inherently limited by the range of models and sample sizes considered, our results illustrate the promise of AFS-based methods like δaδi [4], Jaatha [5], MultiPop [6], and fastsimcoal2 [7] for demographic inference and highlight the importance of collecting large population-level genomic samples for analyses of the AFS, particularly when interest lies in estimating parameter values associated with recent demographic changes.

## Methods

δaδi is distributed as a module written in Python [4]. We simulated datasets as Poisson samples from the expected AFS under the simulated models (using scripts modified from the δaδi user group). Therefore, our datasets meet the assumption of unlinked sites, but empirical data generally will not. For each parameterization and sample size, we set the grid sizes for the finite difference approximation of the solution to the partial differential equation modeling the density of SNPs in each cell of the AFS to [2*n* + 10, 2*n* + 20, and 2*n* + 30], where *n* is the number of diploid individuals sampled per population. We initially simulated 100 datasets for each of the model × parameterization × sample size combinations, for a total of 9800 simulations. However, problems with convergence limited the analysis of these datasets to slightly smaller subsets for both model selection and parameter estimation analyses. Here we present parameter estimation results from 9792 simulated datasets and model selection results from a total of 9757 datasets. Sample sizes, model parameterizations, and simulated demographic models are given below for each population configuration.

### One-population models

We simulated four single-population demographic models (Figure 2), including: 1) constant population size (SNM), 2) exponential population growth (POSG), 3) exponential population decline (negative growth; NEGG), and 4) a bottleneck followed by exponential growth (BG). For each model, we simulated samples of 2, 3, 5, 10, 20, 30, and 50 diploid individuals (4, 6, 10, 20, 40, 60, and 100 chromosomes). The alternative parameterizations for each of the single-population demographic models are given in Table 1. Briefly, models included up to four parameters: *θ*, *η*_*D*_, *η*_*G*_, and *T. θ* is a composite parameter based on the genomic region surveyed for SNPs and is equal to 4*N*_*A*_*μL*, where *N*_*A*_ is the effective size of the ancestral population, *μ* is the neutral rate of mutation per base pair per generation, and *L* is the total number of base pairs surveyed for variation. Population size changes (decline – *η*_*D*_, growth – *η*_*G*_) are specified as proportional changes in effective size relative to *N*_*A*_. Finally, all times in the models (*T*) are measured in units of 2*N*_*A*_ generations. Parameter optimization was carried out using the Broyden-Fletcher-Goldfarb-Shanno (BFGS) method as implemented in the δaδi function ‘optimize_log’ [4]. Upper and lower bounds for parameter optimization steps were set to the following ranges: bottleneck proportion or magnitude of population decline (BG and NEGG models; 0.001-1), magnitude of population growth (BG and POSG models; 1-100), timing of demographic events (BG, POSG, NEGG models; 0.01-5). For some models, we adjusted these ranges for optimization, in order to achieve more rapid convergence of the estimates. For instance, we decreased the lower bound on *T* for the BG model with a recent bottleneck, for sample sizes of 50 individuals. We used a step size of *ε* = 1 × 10^−6^ for all parameter optimization steps. By using the ‘optimize_log’ function, negative parameter estimates were not allowed.

### Two-population models

We simulated three two-population models in our study (Figure 3), including: 1) no divergence (SNM), 2) divergence in isolation (ISO), and 3) divergence with gene flow (IM). For each of these models, we simulated sample sizes of 1, 2, 3, 5, 10, 20, and 30 diploid individuals per population (2, 4, 6, 10, 20, 40, and 60 chromosomes). Issues with convergence of two-population models (ISO and IM) to data simulated under a single-population history (SNM) led us to discard some replicates from the model selection analysis. The ISO model failed to converge (using our criterion) for 20 of the 700 datasets, while the IM model failed in 15 of 700 datasets. Thus the total number of SNM datasets considered in model comparison analyses was reduced to 665 of the 700 simulated replicates. Additionally, eight of the 700 IM datasets with recent divergence (B parameterization) failed to converge, resulting in 692 total datasets analyzed for model selection and parameter estimation. Alternative parameterizations for each model are given in Table 2. Briefly, two-population models included as many as seven parameters: *θ*, *s*, *η*_*1*_, *η*_*2*_, *T*, *m*_*12*_, and *m*_*21*_. The parameters *θ, η*_*1*_, *η*_*2*_, and *T* are defined as in single-population models. The parameter *s* gives the fraction of the ancestral population founding population 1 (1 - *s* gives this proportion for population 2). Migration parameters (*m*_*12*_ and *m*_*21*_) were specified as 2*N*_*A*_*M*_ij_ where *M*_*ij*_ was the proportion of population *i* derived from migrants from population *j* each generation. Parameter optimizations were carried out as in the one-population models. Upper and lower bounds for parameter optimization were set as follows: fraction of ancestral population founding population 1 (IM and ISO models; 0.01-0.99), magnitude of population expansion following divergence (IM and ISO models; 0.5-10), timing of population divergence (IM and ISO models; 0.005-5), and migration rates between populations (IM model; 0.1-20). Similarly to the one-population models, we decreased the lower bounds on some parameters (e.g., *T* and *m*) in an effort to speed convergence for the IM and ISO model fits in datasets with larger sample sizes. Additionally, starting parameter values for the optimization were altered when fitting IM and ISO models to SNM datasets. In all cases, starting points for the optimization were perturbed for each parameter optimization.

### Statistical analysis

Parameter optimization was carried out for each of the models compared for each replicate dataset. Then, the likelihood of the model, given the maximum-likelihood parameter estimates, was calculated. In order to ensure convergence of parameter estimates for each replicate dataset, we iteratively fit each of the models compared until we obtained three parameter optimizations with log-likelihood values within five units of the highest likelihood. Following this process, we used parameter estimates associated with the optimization that resulted in the largest log-likelihood value as our maximum likelihood estimates for a given replicate dataset. Log-likelihoods for the competing models were then used to calculate AIC [21] as

$$ AIC=-2 \log L+2K $$

where 2*K* is a penalty based on the number of parameters (*K*) in a given model. We also calculated Akaike weights, which show the proportional support for each of a series of competing models, using the following equation

$$ {w}_i=\frac{e^{-\frac{1}{2}{\Delta}_{AI{C}_i}}}{{\displaystyle \sum {e}^{-\frac{1}{2}{\Delta}_{AI{C}_i}}}} $$

where Δ_*AICi*_ is the difference between the smallest AIC value and that associated with model *i* [22]. It is important to note that, when the assumption of unlinked SNP loci is violated (as in most empirical datasets), model comparisons based on AIC may be biased [7]. Varin and Vidoni [31] introduced a composite likelihood information criterion to account for this bias, but this correction is not necessary in our simulated data, where SNPs were simulated as unlinked and independent.

We used the median as a measure of central tendency for the distribution of parameter estimates and the RMSE to assess the precision and accuracy of estimates for each model and sample size considered. RMSE for a parameter estimate $\left(\widehat{\theta}\right)$ was calculated as

$$ RMS{E}_{\theta }=\sqrt{\frac{{\displaystyle \sum {\left(\widehat{\theta}-\theta \right)}^2}}{n}} $$

where *θ* is the simulated parameter value and *n* is the number of replicate datasets analyzed. The uncertainty in parameter estimates for each simulated dataset was approximated using the Hessian matrix (Fisher Information Matrix) using modifications of a script from the δaδi user group. We then compared the median uncertainty in parameter estimates across sample sizes and parameterizations.

As an additional measure of the accuracy of inferences drawn from analysis of the AFS, we also assessed the coverage of 95% CI based on estimates of uncertainty from the Hessian matrix. Confidence intervals were constructed as a symmetrical range, extending to ±1.96σ_*HESS*_ around the maximum likelihood parameter estimate, where σ_*HESS*_ is the estimate of the uncertainty in a given parameter estimate obtained from the Hessian matrix (see above). Ideally, these intervals would contain the true (simulated) parameter value in ~95% of the simulated replicates. We were unable to calculate the Hessian matrix for all analyzed datasets, so the results we present for coverage and the median uncertainty for a given sample size are based on the subset of simulated datasets where these estimates were obtained. Our inability to calculate the Hessian matrix appears to be related to the timing of demographic events in our simulations, based on the number of datasets that exhibited these difficulties with recent population size changes (POSGa – 2, POSGb – 25, NEGGa – 1, NEGGb – 9, BGa – 15, BGb – 111) or divergence (ISOa - 1, ISOb - 152 , IMa – 38, IMb – 170). In many of these cases, our convergence criterion may not have insured that our model fits were truly the most likely values. Then, negative entries along the diagonal of the Hessian matrix led to negative variances in the variance-covariance matrix, making uncertainty estimation impossible using the present framework.

### Availability of supporting data

The Python scripts used to generate and analyze datasets for this article, the simulated data sets, and the raw parameter estimates and their associated uncertainties are available online in the LabArchives respository http://dx.doi.org/10.6070/H4GQ6VQ2.

## Additional file

Additional file 1:
**This file contains additional tables and figures not presented in the manuscript.** Figures and tables in this file include: comparisons of parameter estimates from optimizations employing different starting points, comparisons of frequency spectra generated in ms and δaδi, model selection results from the Extreme cases simulations, statistics associated with single-population, two-population, and Extreme cases simulations, and tables of the average number of SNPs in datasets under different model, parameterization, and sample size combinations.
