# Supplementary material for: Sampling strategies for frequency spectrum-based population genomic inference
Source: BMC Evol Biol. 2014 Dec 4;14:254. doi: 10.1186/s12862-014-0254-4 (PMC4269862; doi:10.1186/s12862-014-0254-4)
Supplement: Additional file 1: — This file contains additional tables and figures not presented in the manuscript. Figures and tables in this file include: comparisons of parameter estimates from optimizations employing different starting points, comparisons of frequency spectra generated in ms and δaδi, model selection results from the Extreme cases simulations, statistics associated with single-population, two-population, and Extreme cases simulations, and tables of the average number of SNPs in datasets under different model, parameterization, and sample size combinations. [file 12862_2014_254_MOESM1_ESM.pdf]

Additional file 1

Figure S1. Comparison of *s* estimates for the isolation (ISO) model with recent divergence using different starting points and parameter perturbation settings. Sample sizes are given above each plot (e.g., 2x2 indicates samples of two chromosomes, or one diploid individual, per population). For each sample size, *s* estimates obtained using a starting point of 0.5 and a perturbation fold of 2 ($s_{0.5}$) are plotted versus the same dataset analyzed with a starting point of 0.25 and a perturbation fold of 1 ($s_{0.25}$). The smaller starting point led to a downward bias for smaller sample sizes, as evidenced by the clustering of points to the left of figures with sample sizes smaller than 20 diploid individuals per population.

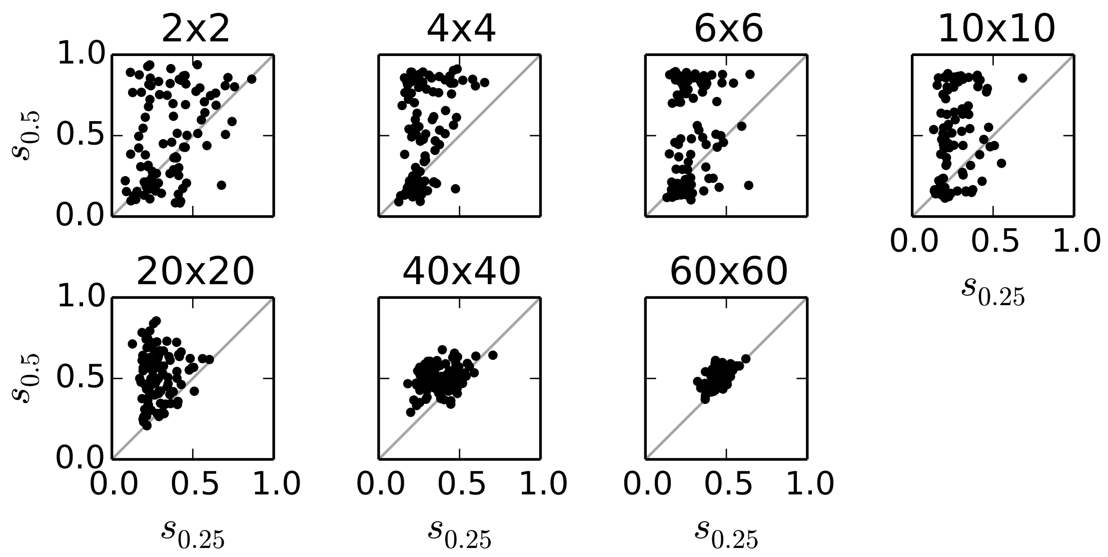

Figure S2. Comparison between allele frequency spectra simulated in δaδi (Gutenkunst et al. 2009) and in the coalescent simulator ms (Hudson 2002) for a model of ancient ($T = 1.5$) population growth (POSG; Table S3) with $n = 30$ diploid individuals sampled. The top panel shows the frequency spectrum obtained under each approach (ms – blue, δaδi – red), while the bottom shows Anscombe Poisson residuals for each cell of the AFS. The ms spectrum was generated by running 10^8 ms simulations with θ=1, taking the mean of each entry across those simulations, and multiplying each entry by 4500 to match θ for the corresponding δaδi simulation using the parameters in Table S3. A similar procedure was followed to generate Figures S3 and S4.

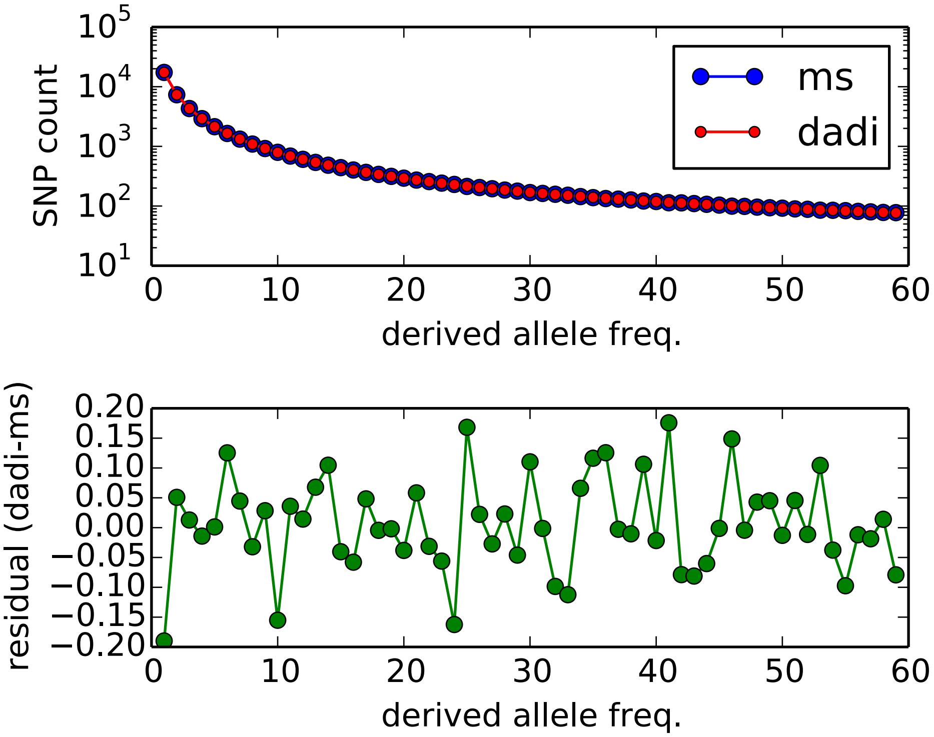

Figure S3. Comparison between allele frequency spectra simulated in δaδi (Gutenkunst et al. 2009) and in the coalescent simulator ms (Hudson 2002) for a model of population growth (POSG; Table S4) with n = 1351 diploid individuals sampled. The top panel shows the frequency spectrum obtained under each approach (ms – blue, δaδi – red), while the bottom shows Anscombe Poisson residuals for each cell of the AFS.

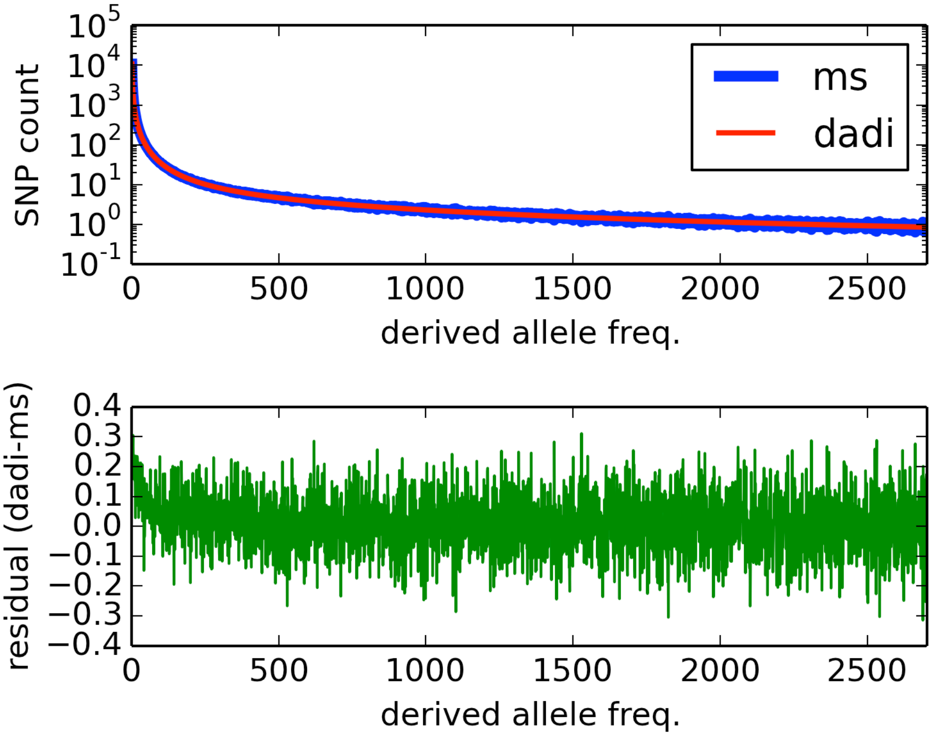

Figure S4. Comparison between allele frequency spectra simulated in δaδi (Gutenkunst et al. 2009) and in the coalescent simulator ms (Hudson 2002) for a model of very recent ($T = 0.005$) divergence (ISO; Table S5) with $n = 30$ diploid individuals sampled. The upper panels show the frequency spectra, while the lower panels show the Anscombe Poisson residuals between dadi and ms.

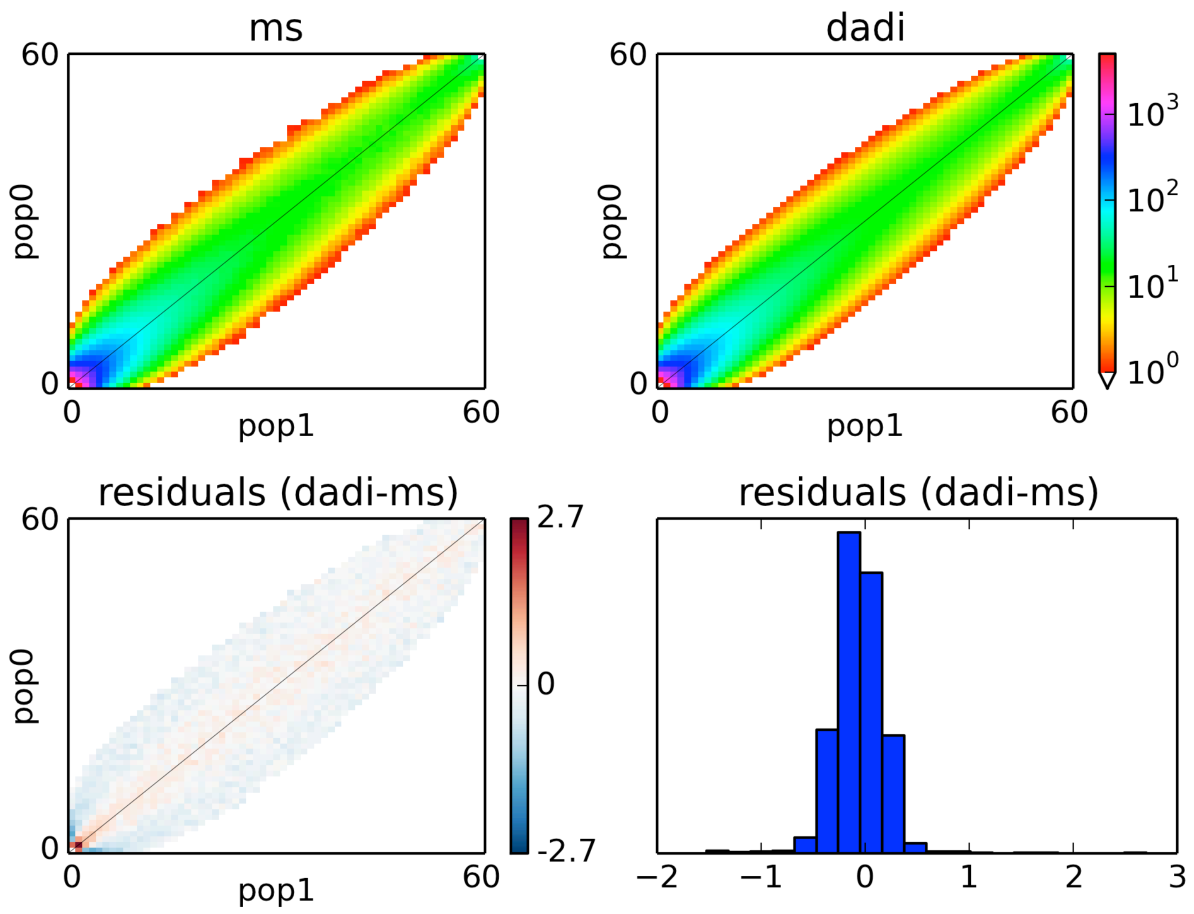

Figure S5. Model selection results for single-population datasets (n = 86 datasets) simulated with older demographic events (T = 1.5). Model parameters are slightly modified from the A parameterization in Table 1. The distribution of Akaike weights in favor of each model is shown for data simulated under the four single-population demographic models. All datasets consisted of n = 30 diploid individuals.

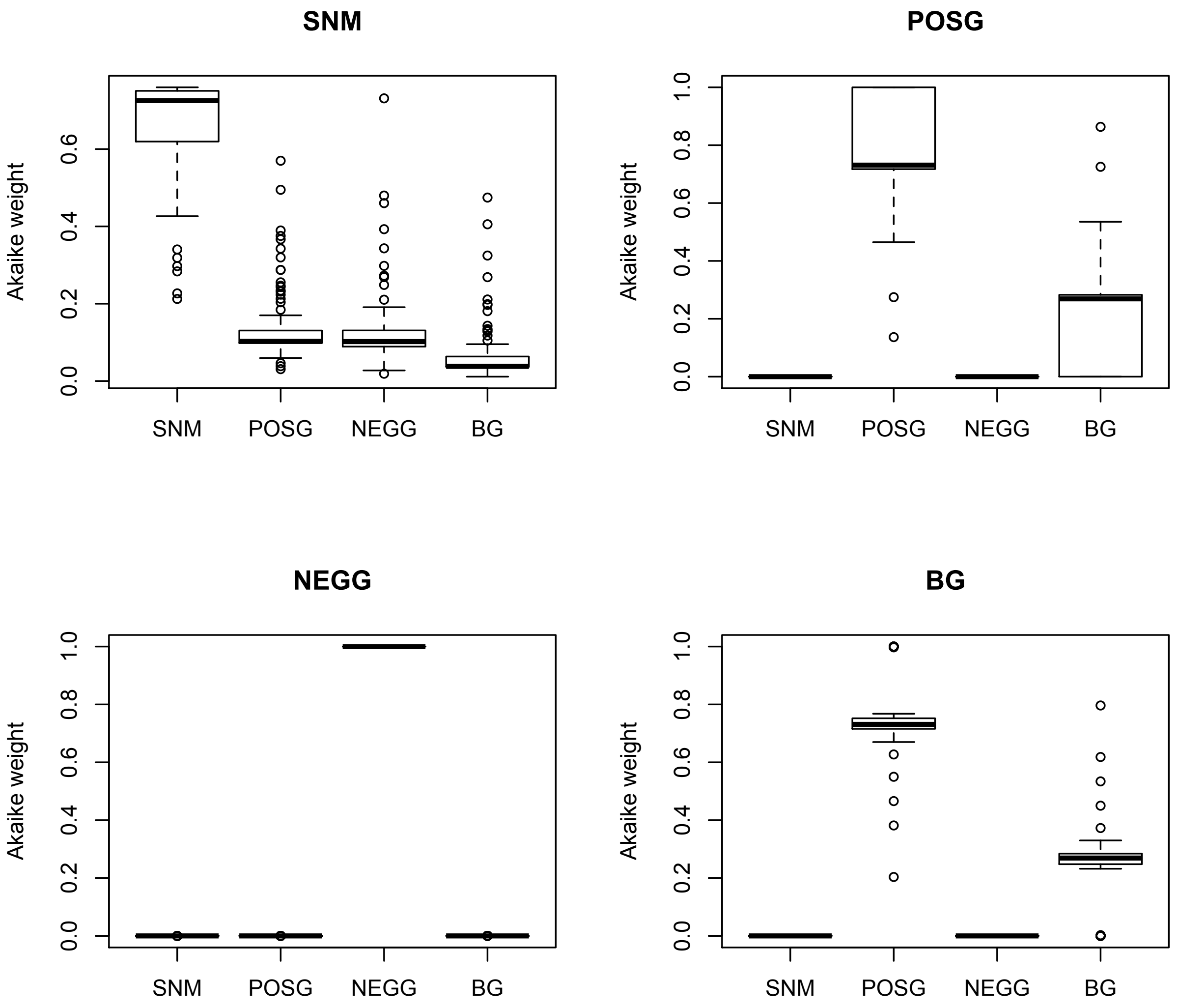

Figure S6. Model selection results for single-population datasets (n = 100 datasets) with large sample sizes (n = 1351 diploid individuals). Model parameters are slightly modified from the A parameterization in Table 1. The distribution of Akaike weights in favor of each model is shown for data simulated under the four single-population demographic models.

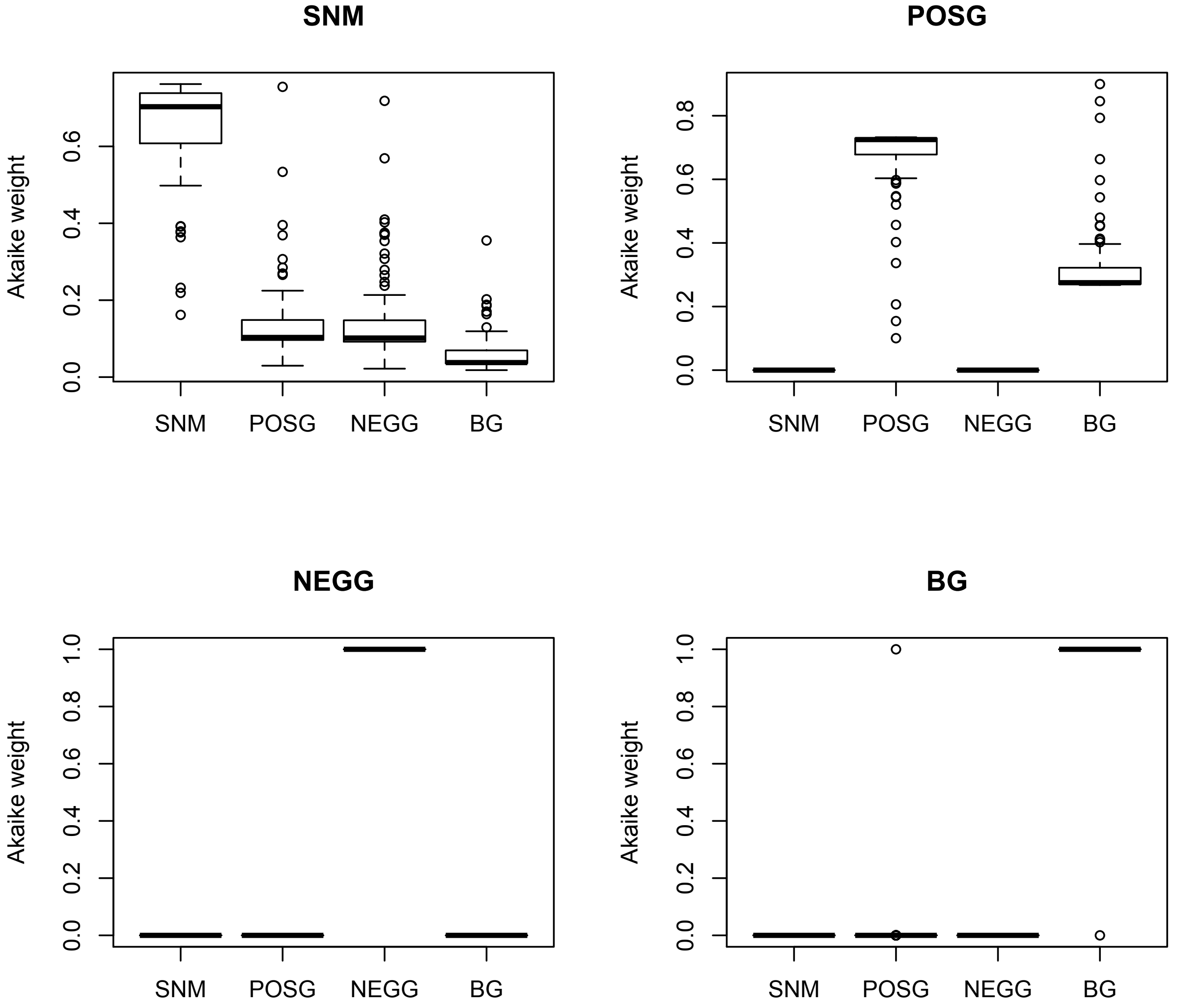

Figure S7. Model selection results for two-population datasets (n = 100 datasets) with very recent divergence (T = 0.005) and sample sizes of n = 30 diploid individuals per population. Model parameters are slightly modified from the A parameterization in Table 2. The distribution of Akaike weights in favor of each model is shown for data simulated under the four single-population demographic models.

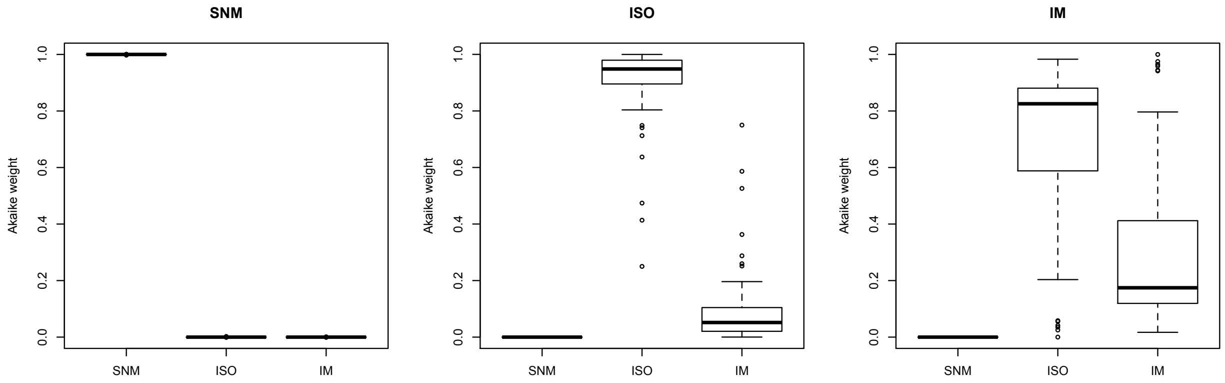

Table S1. Statistics associated with single-population datasets. Median estimates, median uncertainties, RMSE, and the proportional coverage of 95% confidence intervals for all model parameters, models, and sample sizes are given.

Model	Parameter	Simulated Value	n	Median Estimate	Median Uncertainty	RMSE	Coverage of 95% CI	Simulated Value	n	Median Estimate	Median Uncertainty	RMSE	Coverage of 95% CI
SNM	θ	10000	2	10002.49	74.33	69.64	0.94	NA	--	--	--	--	
			3	9997.60	66.54	66.62	0.93		--	--	--	--	
			5	10007.04	59.73	56.30	0.96		--	--	--	--	
			10	9995.64	53.20	47.56	0.98		--	--	--	--	
			20	10001.68	48.53	46.10	0.95		--	--	--	--	
			30	10003.61	46.34	39.49	0.97		--	--	--	--	
			50	10003.10	43.96	44.34	0.94		--	--	--	--	
POSG	θ	6000	2	5993.26	146.61	215.66	1.00	6000	2	5923.41	164.99	427.81	0.98
			3	5986.41	99.60	99.59	0.94		3	5945.61	88.15	279.28	0.96
			5	6014.06	77.54	80.15	0.95		5	5984.77	69.86	216.83	0.97
			10	6003.92	65.10	73.06	0.92		10	5986.54	55.73	60.26	0.97
			20	6005.90	58.75	61.95	0.93		20	6002.47	49.71	53.41	0.92
			30	5996.26	56.87	59.62	0.93		30	6004.14	46.72	49.88	0.94
			50	6009.10	55.01	51.39	0.96		50	5993.61	44.21	48.31	0.95
	η_G	5	2	4.95	4.53	36.07	0.77	2.5	2	1.25	1.18	20.68	0.69
			3	5.01	2.89	19.05	0.81		3	1.24	0.90	28.58	0.54
			5	5.22	1.30	1.59	0.95		5	1.38	0.85	17.58	0.58
			10	4.98	0.41	0.43	0.95		10	2.04	1.56	12.40	0.75
			20	5.00	0.20	0.20	0.95		20	2.25	0.87	4.02	0.88
			30	4.99	0.14	0.13	0.96		30	2.54	0.72	2.72	0.89
			50	5.00	0.10	0.10	0.95		50	2.49	0.37	2.45	0.92
	T	0.25	2	0.260	0.212	0.311	0.82	0.025	2	0.374	0.865	2.178	1.00
			3	0.257	0.089	0.090	0.93		3	0.122	0.471	1.964	0.98
			5	0.239	0.041	0.038	0.95		5	0.070	0.163	1.148	1.00
			10	0.254	0.021	0.022	0.96		10	0.036	0.050	0.236	0.88
			20	0.251	0.013	0.014	0.93		20	0.028	0.017	0.221	0.89
			30	0.251	0.011	0.011	0.94		30	0.025	0.010	0.223	0.95
			50	0.251	0.009	0.009	0.98		50	0.025	0.006	0.224	0.96

**NEGG**	***θ***	**29000**	2	28105.22	3416.19	6362.93	0.87	**29000**	2	29121.12	349.15	411.59	0.96
			3	29038.60	1730.54	5903.26	0.97		3	29077.00	181.63	242.64	0.94
			5	28771.96	993.48	1194.02	0.89		5	29062.56	155.21	164.73	0.97
			10	28979.46	744.54	864.28	0.92		10	28989.07	133.26	140.42	0.94
			20	28982.91	621.95	575.11	0.95		20	29016.82	122.51	122.04	0.96
			30	29014.32	590.10	637.83	0.92		30	29014.46	116.74	116.48	0.97
			50	29141.43	556.60	572.50	0.97		50	29034.66	112.89	122.15	0.93
	η_D	**0.1**	2	0.092	0.020	0.021	0.91	**0.25**	2	0.53	0.27	0.45	0.72
			3	0.101	0.008	0.008	0.93		3	0.26	0.21	0.31	0.80
			5	0.099	0.005	0.004	1.00		5	0.23	0.15	0.23	0.80
			10	0.100	0.003	0.003	0.96		10	0.24	0.08	0.16	0.82
			20	0.100	0.002	0.002	0.98		20	0.25	0.03	0.15	0.94
			30	0.100	0.002	0.002	0.98		30	0.25	0.02	0.15	0.97
			50	0.100	0.002	0.002	0.96		50	0.25	0.01	0.15	0.92
	T	**0.25**	2	0.218	0.097	0.093	0.87	**0.025**	2	0.075	0.239	0.236	0.99
			3	0.251	0.041	0.046	0.97		3	0.027	0.066	0.215	0.97
			5	0.242	0.022	0.023	0.92		5	0.022	0.031	0.217	0.86
			10	0.251	0.013	0.015	0.90		10	0.024	0.012	0.226	0.84
			20	0.251	0.009	0.009	0.95		20	0.025	0.005	0.225	0.93
			30	0.252	0.008	0.009	0.92		30	0.025	0.003	0.225	0.93
			50	0.251	0.007	0.007	0.94		50	0.025	0.002	0.224	0.92
**BG**	***θ***	**10000**	2	10280.39	1531.14	2498.98	0.92	**10000**	2	10083.41	144.01	449.93	0.93
			3	10150.05	583.51	1320.25	0.86		3	10043.09	109.27	169.62	0.94
			5	10062.26	339.46	436.98	0.82		5	10031.89	96.04	97.19	0.94
			10	9993.25	167.91	239.15	0.84		10	10014.87	80.69	78.50	0.97
			20	10007.37	114.54	227.96	0.70		20	10000.51	71.32	88.81	0.95
			30	10037.95	105.67	223.35	0.71		30	10008.67	70.97	122.70	0.98
			50	9974.56	90.36	214.03	0.67		50	10004.41	65.79	116.08	0.92
	η_D	**0.1**	2	0.08	0.03	0.05	0.70	**0.25**	2	0.33	0.17	0.43	0.71
			3	0.10	0.02	0.02	0.82		3	0.22	0.07	0.37	0.56
			5	0.10	0.01	0.01	0.83		5	0.19	0.10	0.33	0.62
			10	0.10	0.00	0.01	0.80		10	0.25	0.17	0.40	0.59
			20	0.10	0.00	0.01	0.60		20	0.26	0.13	0.30	0.59

			30	0.10	0.00	0.01	0.67		30	0.30	0.09	0.32	0.68
			50	0.10	0.00	0.01	0.51		50	0.26	0.04	0.32	0.85
	η_G	**5**	2	4.27	5.56	20.41	0.78	**2.5**	2	1.96	3.42	23.05	0.83
			3	4.39	1.83	12.84	0.72		3	3.20	4.38	23.18	0.82
			5	4.95	0.87	1.59	0.75		5	2.96	3.11	25.72	0.76
			10	4.98	0.30	0.46	0.77		10	1.86	1.71	21.79	0.56
			20	4.98	0.16	0.23	0.87		20	2.32	1.38	19.56	0.67
			30	4.99	0.13	0.20	0.76		30	2.02	0.79	18.14	0.62
			50	4.99	0.10	0.17	0.75		50	2.46	0.48	2.69	0.82
	T	**0.25**	2	0.246	0.051	0.066	0.73	**0.025**	2	0.062	0.084	0.258	0.75
			3	0.255	0.023	0.042	0.78		3	0.040	0.027	0.213	0.70
			5	0.250	0.010	0.017	0.76		5	0.025	0.019	0.214	0.72
			10	0.250	0.005	0.007	0.86		10	0.026	0.015	0.208	0.76
			20	0.249	0.004	0.005	0.83		20	0.026	0.012	0.215	0.72
			30	0.250	0.003	0.004	0.86		30	0.029	0.009	0.225	0.73
			50	0.251	0.003	0.004	0.75		50	0.026	0.004	0.405	0.86

Table S2. Statistics associated with two-population datasets. Median estimates, median uncertainties, RMSE, and the proportional coverage of 95% confidence intervals for all model parameters models, and sample sizes are given.

Model	Parameter	Simulated Value	*n*	Median Estimate	Median Uncertainty	RMSE	Coverage of 95% CI	Simulated Value	*n*	Median Estimate	Median Uncertainty	RMSE	Coverage of 95% CI
**SNM**	θ	**10000**	1	10017.97	74.59	75.24	0.96	**NA**	--	--	--	--	--
			2	10007.93	62.62	71.94	0.95		--	--	--	--	--
			3	9996.10	57.93	57.29	0.96		--	--	--	--	--
			5	10005.86	53.39	47.90	0.97		--	--	--	--	--
			10	10005.07	48.64	49.07	0.94		--	--	--	--	--
			20	10005.58	45.01	41.01	0.96		--	--	--	--	--
			30	9989.46	43.21	45.61	0.93		--	--	--	--	--
**ISO**	θ	**7250**	1	7250.41	99.19	96.42	0.96	**7250**	1	7235.79	94.11	84.08	0.97
			2	7263.58	72.71	80.20	0.94		2	7238.79	68.72	60.55	0.98
			3	7241.85	65.64	66.38	0.94		3	7256.19	61.28	61.59	0.98
			5	7246.54	59.92	55.61	0.96		5	7253.37	54.76	56.03	0.94
			10	7245.33	55.14	55.39	0.97		10	7256.28	49.13	52.79	0.96
			20	7245.45	52.22	54.32	0.95		20	7241.88	44.95	44.70	0.97
			30	7260.21	51.09	53.79	0.94		30	7246.88	43.05	42.86	0.94
	s	**0.5**	1	0.47	0.18	0.22	0.67	**0.5**	1	0.36	0.47	0.23	0.81
			2	0.51	0.07	0.07	0.91		2	0.25	0.20	0.24	0.58
			3	0.50	0.04	0.05	0.87		3	0.25	0.19	0.24	0.67
			5	0.50	0.03	0.02	0.97		5	0.23	0.11	0.25	0.41
			10	0.50	0.02	0.02	0.94		10	0.27	0.11	0.23	0.52
			20	0.50	0.01	0.01	0.93		20	0.38	0.07	0.16	0.55
			30	0.50	0.01	0.01	0.92		30	0.45	0.04	0.07	0.81
	η_1	**1**	1	1.06	0.68	3.16	0.82	**1**	1	2.46	5.85	4.01	0.99
			2	0.98	0.16	0.17	0.92		2	2.76	4.40	3.13	0.97
			3	1.00	0.10	0.10	0.92		3	2.66	3.26	3.02	0.96
			5	1.00	0.06	0.05	0.98		5	2.67	2.28	3.13	0.96
			10	1.01	0.03	0.04	0.96		10	2.03	0.94	1.63	0.90
			20	1.00	0.02	0.02	0.94		20	1.31	0.23	0.70	0.78
			30	1.00	0.02	0.02	0.96		30	1.07	0.10	0.14	0.89

	***η*₂**	**1**	1	0.95	0.53	1.39	0.76	**1**	1	0.89	1.74	1.98	0.97
			2	1.01	0.16	0.16	0.92		2	0.68	0.41	0.39	0.93
			3	1.01	0.10	0.12	0.87		3	0.69	0.26	0.34	0.79
			5	0.99	0.06	0.05	1.00		5	0.71	0.14	0.32	0.55
			10	1.00	0.03	0.04	0.95		10	0.72	0.12	0.29	0.46
			20	1.00	0.02	0.02	0.95		20	0.84	0.09	0.21	0.56
			30	1.00	0.02	0.02	0.98		30	0.94	0.07	0.10	0.78
	T	**0.25**	1	0.247	0.013	0.013	0.93	**0.025**	1	0.026	0.010	0.008	0.91
			2	0.249	0.007	0.007	0.94		2	0.025	0.004	0.004	0.97
			3	0.250	0.005	0.005	0.96		3	0.024	0.003	0.003	0.96
			5	0.250	0.004	0.003	0.97		5	0.025	0.002	0.002	0.94
			10	0.250	0.003	0.003	0.97		10	0.025	0.001	0.001	0.95
			20	0.250	0.002	0.002	0.95		20	0.025	0.001	0.001	0.88
			30	0.249	0.002	0.002	0.96		30	0.025	0.000	0.000	0.94
**IM**	***θ***	**7250**	1	7246.28	203.39	305.88	0.96	**7250**	1	7221.27	160.54	144.97	0.93
			2	7264.36	88.28	88.09	0.97		2	7238.68	98.11	79.05	0.98
			3	7265.99	74.06	77.11	0.93		3	7250.48	74.04	78.58	0.97
			5	7244.66	64.38	60.66	0.96		5	7244.09	58.55	62.06	0.97
			10	7239.43	57.56	54.82	0.96		10	7244.47	50.20	60.79	0.95
			20	7254.67	53.80	58.82	0.91		20	7252.22	45.14	47.09	0.95
			30	7251.44	52.29	56.89	0.93		30	7266.62	43.41	41.67	0.96
	s	**0.5**	1	0.36	0.31	0.26	0.67	**0.5**	1	0.47	0.46	0.27	0.71
			2	0.51	0.12	0.14	0.84		2	0.50	0.35	0.25	0.78
			3	0.51	0.07	0.07	0.92		3	0.51	0.25	0.26	0.67
			5	0.50	0.04	0.04	0.95		5	0.56	0.18	0.23	0.64
			10	0.50	0.02	0.02	0.93		10	0.52	0.15	0.17	0.77
			20	0.50	0.02	0.02	0.94		20	0.53	0.08	0.10	0.88
			30	0.50	0.01	0.03	0.93		30	0.50	0.05	0.07	0.86
	***η*₁**	**1**	1	1.01	0.90	1.85	0.88	**1**	1	1.27	1.61	2.73	1.00
			2	1.00	0.20	0.27	0.90		2	1.06	1.52	2.51	0.97
			3	0.98	0.10	0.12	0.92		3	1.09	0.91	2.74	0.83
			5	1.01	0.06	0.06	0.98		5	0.97	0.47	2.55	0.82
			10	1.01	0.03	0.04	0.93		10	1.01	0.33	1.62	0.85

			20	1.00	0.02	0.02	0.94		20	0.99	0.14	0.16	0.90
			30	1.00	0.02	0.02	0.94		30	0.97	0.09	0.11	0.86
			1	0.85	0.25	0.54	0.79		1	1.05	2.19	2.03	0.95
			2	1.00	0.19	0.40	0.91		2	0.93	1.28	2.21	0.93
			3	1.01	0.11	0.12	0.97		3	0.99	1.05	2.27	0.88
	**η_2**	**1**	5	1.00	0.06	0.06	0.97	**1**	5	1.03	0.73	1.13	0.80
			10	1.00	0.04	0.04	0.88		10	0.93	0.36	0.71	0.81
			20	1.00	0.02	0.03	0.92		20	1.08	0.18	0.30	0.93
			30	1.00	0.02	0.05	0.90		30	1.02	0.10	0.16	0.89
			1	0.279	0.155	0.326	0.90		1	0.027	0.045	0.064	0.90
			2	0.239	0.034	0.126	0.86		2	0.030	0.024	0.022	0.97
			3	0.244	0.022	0.027	0.92		3	0.026	0.016	0.042	0.96
	**T**	**0.25**	5	0.254	0.013	0.015	0.92	**0.025**	5	0.027	0.009	0.032	0.92
			10	0.251	0.008	0.008	0.96		10	0.026	0.005	0.053	0.90
			20	0.250	0.006	0.008	0.90		20	0.024	0.002	0.002	0.82
			30	0.251	0.006	0.008	0.95		30	0.025	0.001	0.002	0.86
			1	1.54	1.26	0.96	0.79		1	10.58	19.27	7.79	0.88
			2	0.96	0.35	0.38	0.88		2	12.26	15.98	7.23	0.93
			3	0.94	0.18	0.20	0.95		3	7.57	11.11	6.53	0.87
	**m_{12}**	**1**	5	1.00	0.10	0.10	0.95	**10**	5	9.91	7.91	6.26	0.81
			10	0.99	0.05	0.05	0.95		10	9.09	4.03	4.92	0.77
			20	1.00	0.04	0.04	0.94		20	9.34	1.82	2.57	0.75
			30	1.00	0.03	0.04	0.96		30	10.22	1.23	2.12	0.78
			1	0.84	1.13	0.72	0.87		1	1.56	21.17	6.66	0.97
			2	0.94	0.35	0.37	0.95		2	2.14	15.34	6.41	0.95
			3	1.01	0.18	0.22	0.85		3	1.77	12.94	6.24	0.84
	**M_{21}**	**1**	5	0.99	0.10	0.11	0.88	**1**	5	1.42	7.69	5.62	0.85
			10	1.00	0.05	0.05	0.95		10	2.01	4.02	4.05	0.88
			20	1.00	0.04	0.04	0.92		20	0.51	1.59	1.09	0.97
			30	1.00	0.03	0.04	0.85		30	0.55	0.92	0.84	0.92

Table S3. Statistics associated with additional simulations of single-population models with more ancient events ($T = 1.5$). Simulated parameter values, median estimates, and RMSE are given for all parameters of the four single-population models. Datasets consisted of $n = 30$ diploid individuals. Model parameters are slightly modified from the A parameterization in Table 1.

**Model**	**Parameter**	**Simulated Value**	**Median Estimate**	**RMSE**
SNM	θ	10,700	10,690.1	34.9
POSG	θ	4500	4480.7	28.7
	η_G	5	5.017	0.0123
	T	1.5	1.506	0.0711
NEGG	θ	91,500	106,108.7	73,649.4
	η_D	0.1	0.086	0.3092
	T	1.5	1.322	0.6167
BG	θ	6700	9720.6	2602.6
	η_D	0.1	0.052	0.0989
	η_G	5	3.375	1.2565
	T	1.5	1.100	1.0873

Table S4. Statistics associated with additional simulations of single-population models with much larger sample size (n = 1351 individuals, as for European samples analyzed by Tennessen et al. 2012). Simulated parameter values, median estimates, and RMSE are given for all parameters of the four single-population models. Datasets consisted of n = 1351 diploid individuals. Model parameters are slightly modified from the A parameterization in Table 1.

**Model**	**Parameter**	**Simulated Value**	**Median Estimate**	**RMSE**
SNM	θ	5900	5897.9	43.6
POSG	θ	2300	2295.2	11.3
	η_G	5	5.006	0.0586
	T	0.25	0.250	0.0037
NEGG	θ	23,600	23,592.4	268.8
	η_D	0.1	0.100	0.0022
	T	0.25	0.250	0.0033
BG	θ	3000	3021.7	77.8
	η_D	0.1	0.099	0.0071
	η_G	5	4.964	0.0020
	T	0.25	0.250	0.0020

Table S5. Statistics associated with additional simulations of two-population models with much more recent divergence ($T = 0.005$, $0.01N_A$ generations). Simulated parameter values, median estimates, and RMSE are given for all parameters of the four single-population models. Datasets consisted of $n = 30$ diploid individuals per population. Model parameters are slightly modified from the A parameterization in Table 2.

**Model**	**Parameter**	**Simulated Value**	**Median Estimate**	**RMSE**
SNM	θ	9300	9295.6	15.8
ISO	θ	9200	9211.0	43.3
	s	0.5	0.500	0.1829
	η_1	1	1.018	0.1248
	η_2	1	0.991	0.1754
	T	0.005	0.00487	0.00009
IM	θ	9200	9210.6	28.1
	s	0.5	0.434	0.1689
	η_1	1	1.112	0.1502
	η_2	1	0.891	0.0855
	T	0.005	0.00493	0.00009
	m_{12}	1	0.766	4.6695
	m_{21}	1	0.692	0.7105

Table S6. Average numbers of single nucleotide polymorphism (SNP) loci in simulated datasets. Simulated theta values were set to give ~50,000 SNP loci in the largest datasets for "easy" parameterizations (e.g. IMa, POSGa, NEGGa, BGa), and this parameter value was used for all simulations of that model ("easy" and "hard" parameterizations, all sample sizes).

1 population models

Sample Size	SNM	POSGa	POSGb	NEGGa	NEGGb	BGa	BGb
4	18,109.9	12,292.7	10,979.3	26,524.6	50,817.1	13,721.4	17,928.0
6	22,560.4	15,641.5	13,717.0	31,994.3	62,955.9	17,268.2	22,322.6
10	28,072.1	20,189.7	17,122.5	37,420.6	77,298.8	21,887.1	27,620.2
20	35,289.9	27,299.0	21,732.4	42,915.8	94,903.5	29,140.3	34,565.6
40	42,457.2	35,924.6	26,565.9	46,916.8	110,257.0	38,453.4	41,238.3
60	46,607.0	41,939.8	29,545.4	48,811.7	117,904.4	45,246.6	45,237.8
100	51,764.6	50,428.0	33,448.0	50,863.3	126,336.0	55,472.2	50,307.6

2 population models

Sample Size (per pop.)	SNM	ISOa	ISOb	IMa	IMb
2	18,033.5	14,867.9	13,274.7	14,651.0	13,252.8
4	25,544.8	20,980.8	18,796.2	20,790.8	18,789.8
6	29,769.8	24,678.1	21,991.9	24,525.3	21,966.8
10	35,131.4	29,626.4	26,061.5	29,534.4	25,984.3
20	42,305.5	36,994.3	31,715.6	36,885.0	31,623.2
40	49,407.3	45,010.5	37,703.7	44,961.6	37,524.6
60	53,466.5	49,979.4	41,425.8	49,889.6	41,250.3
